# Retinoic Acid Upregulates Ret and Induces Chain Migration and Population Expansion in Vagal Neural Crest Cells to Colonise the Embryonic Gut

**DOI:** 10.1371/journal.pone.0064077

**Published:** 2013-05-22

**Authors:** Johanna E. Simkin, Dongcheng Zhang, Benjamin N. Rollo, Donald F. Newgreen

**Affiliations:** Embryology Laboratory, Murdoch Children’s Research Institute, Royal Children’s Hospital, Parkville VIC, Australia; School of Biomedical Sciences, The University of Queensland, Australia

## Abstract

Vagal neural crest cells (VNCCs) arise in the hindbrain, and at (avian) embryonic day (E) 1.5 commence migration through paraxial tissues to reach the foregut as chains of cells 1–2 days later. They then colonise the rest of the gut in a rostrocaudal wave. The chains of migrating cells later resolve into the ganglia of the enteric nervous system. In organ culture, E4.5 VNCCs resident in the gut (termed enteric or ENCC) which have previously encountered vagal paraxial tissues, rapidly colonised aneural gut tissue in large numbers as chains of cells. Within the same timeframe, E1.5 VNCCs not previously exposed to paraxial tissues provided very few cells that entered the gut mesenchyme, and these never formed chains, despite their ability to migrate in paraxial tissue and in conventional cell culture. Exposing VNCCs *in vitro* to paraxial tissue normally encountered *en route* to the foregut conferred enteric migratory ability. VNCC after passage through paraxial tissue developed elements of retinoic acid signalling such as Retinoic Acid Binding Protein 1 expression. The paraxial tissue's ability to promote gut colonisation was reproduced by the addition of retinoic acid, or the synthetic retinoid Am80, to VNCCs (but not to trunk NCCs) in organ culture. The retinoic acid receptor antagonist CD 2665 strongly reduced enteric colonisation by E1.5 VNCC and E4.5 ENCCs, at a concentration suggesting RARα signalling. By FACS analysis, retinoic acid application to vagal neural tube and NCCs *in vitro* upregulated Ret; a Glial-derived-neurotrophic-factor receptor expressed by ENCCs which is necessary for normal enteric colonisation. This shows that early VNCC, although migratory, are incapable of migrating in appropriate chains in gut mesenchyme, but can be primed for this by retinoic acid. This is the first instance of the characteristic form of NCC migration, chain migration, being attributed to the application of a morphogen.

## Introduction

The enteric nervous system (ENS) is produced largely from vagal level neural crest cells (VNCCs) arising adjacent to somites (s) 1–7 [1], [2]. VNCCs commence migrating from the avian neural tube at embryonic day (E) 1.5 (∼10 somite stage), moving ventrally over and through the paraxial somites towards and into the foregut by E2.5–3 [3]. VNCCs colonise the remaining midgut and hindgut in a rostro-caudal wave of migration, reaching the distal midgut by about E4.5–5 and the distal hindgut by E7.5–8 [4], [5]. Once in the gut these cells are commonly referred to as enteric or ENCCs, and migrate as distinctive chains [6], [7]. ENCCs subsequently differentiate and consolidate into aggregates to create the mature ENS [8]. This developmental process is broadly conserved in vertebrates [9].

As well as chain migration, a determinate of complete colonisation of the growing intestine is continued expansion of the ENCC population [10] via a process termed frontal expansion [11]–[13]. This proliferation is contributed to by mitogenic signals to ENCCs from glial derived neurotrophic factor (GDNF), a growth factor expressed by the gut mesenchyme as early as E3 (HH18) [14]. The cognate receptor Ret is expressed by VNCCs [15] and ENCCs [16], and this signalling pathway induces survival, proliferation, differentiation and chemoattraction to favour migration [17], [18]. This growth factor control scheme is consistent with constitutively activated Ret (MEN 2B mutation) producing ENS hyperplasia in humans [19], [20], and conversely with GDNF+/− mice exhibiting lower ENS density [21], [22].

Retinoic acid (RA) signalling plays many significant roles in development [23]. The small lipophillic RA molecules can be manufactured within the target cell, or enter the cell by diffusion. RA affects ENCC migration with excess RA delivered via maternal injection at mouse E9.5 (equivalent to avian E2.5) producing delayed colonisation of the distal bowel at E12.5 [24]. Decreased RA signalling in retinaldehyde dehydrogenase 2 (*Raldh2)* deficient mice produces an aganglionic bowel phenotype, which can be partially rescued with all-trans-RA treatment of the pregnant mother [25], [26]. However it is not clear whether these effects on the ENS are direct or are due to effects on the gut which interfere with ENS development secondarily.

Indicators of RA synthesis and signalling are present in the somitic mesoderm and foregut endoderm, that is, near the VNCC early migration path. Retinoic acid receptor α (RAR-α) expression is restricted mostly to vagal level neural tube, foregut endoderm and somites at 13 somite stage, [27], [28]. RAR-β is expressed in foregut endoderm and neural tube [27]. Mice with inactivated RARα and β genes show normal initial migration of post-otic (that is, vagal level) NCCs, suggesting these genes are not required for initial migration [29], but later NC patterning was disrupted. RA metabolizing enzyme CYP26A1 is expressed in the vagal level dorsal neural tube/NCCs at 10 somites age, and in the anterior foregut at E2 synchronous with VNCC migration into the foregut [30]. CYP26A1 can metabolize RA into several inactive forms, or into the biologically active product *4-oxo-RA* [31], [32]. The over expression of CYP26A1 can promote growth, proliferation, invasion and migration via upregulation of genes coding for c-Myc and matrix metalloproteases [33], which are molecules of known importance in NCC migration [34], [35]. LacZ reporter assays show murine foregut endoderm with RA binding site retinoic acid response element activity and *Raldh2* transcripts in embryos at times corresponding to avian E1.5 and E2.5 [25], [36], [37]. *Raldh2* mutants display a severely disrupted posterior pharyngeal region [25], [38]–[40]. RA is expressed by mesoderm [41]–[43] such as the presomitic mesoderm at hindbrain level and lateral plate mesoderm, as indicated by Raldh2 expression and by null mutant phenotypes. RA produced locally acts over a considerable distance to affect adjacent tissues such as posterior neuroectoderm, posterior foregut endoderm, and dorsal endoderm [44], [45].

Cardiac (c) NCCs arise from the otic vesicle to s4 level and so share an overlapping anatomical origin with the VNCCs. RA disruption produces a cNCC ablation phenotype with disruptions in the cardiac outflow tract and vagus nerve [46]–[50]. RA also regulates P*hox2b* expression in cNCCs, a transcription factor of known importance in ENCC development [51]. Previous studies have used Retinoic Acid Binding Protein 1 (CRABP-1) *in situ* probes to label migratory cNCCs [46]. CRABP-1 binds both all-trans-retinol and all-trans-retinaldehyde, and presents RA to metabolising enzymes (e.g. CYP26A1). RA application is strongly linked to increases in Ret expression and neural morphology in both enteric and other cell types, including NC-derived neuroblastoma [52], [53]. Notably, a study exposing ENCCs (p75^NTR^ selected from E12.5 mouse gut) to RA plus GDNF showed a delayed increase in Ret expression in culture [16]. Taken together, there is strong evidence that VNCC experience and respond to RA signalling, and that tissues in the vagal neighbourhood produce and respond to RA.

This study investigates the ability of NCC to colonise gut, the role of paraxial tissue on this, and the impact of RA exposure during VNCC initial invasion into gut tissue. We propose that the change from “VNCC” to “ENCC” represents a real functional difference rather than terminological convenience, is accomplished at least in part by exposure to RA normally derived from adjacent tissues, and is marked by increased expression of Ret and by the gain of ability to colonise the gut mesenchyme in large numbers as chains of cells.

## Materials and Methods

### Quail Embryo and Gut Tissue Wholemount and Culture Immunolabeling

E2.5 quail embryos (*Coturnix coturnix;* Lago Game, Melbourne) were fixed in 4% PFA overnight, and dissected with tungsten needles sagittally for wholemount immunolabelling. Embryos were stained overnight with primary antibodies against E/C8 (avian axonal marker,1/10, mouse IgM, Developmental Studies Hybridoma Bank, Iowa City, IA), E-cadherin (endoderm marker, 1/200, mouse IgG, Transduction Lab, KY), and SoxE (NCC marker, 1/2500, rabbit IgG, provided by Dr Craig Smith [54]). Antibodies were dissolved in PBS with 1% horse serum (CSL, Aust.) and 0.1% Triton X-100 (Sigma, Aust.). After extensive washes in PBS, secondary and tertiary labels goat anti-mouse IgGγ: FITC (Zymed, CA), donkey anti-mouse IgM: Texas Red, donkey anti-rabbit:biotin, and streptavidin-AMCA, (Jackson Immunoresearch, PA) were then applied. Specimens were mounted in Vectashield (Vector Laboratories, Inc., CA) between two coverslips with coverslip chips as spacers. For transverse immunolabelling, E2.75–3 quail embryos were fixed then cut manually into 2-somite wide slices from the otocyst to the anterior intestinal portal. Sections were stained overnight with CRABP-1 antibody (1/250, mouse IgG, Abcam) and NCCs were detected using rabbit SoxE antibody as above. Secondary antibodies (anti-mouse IgG: Alexa 488 conjugated; anti-rabbit IgG: Alexa 594 conjugated, Molecular Probes/InVitrogen, OR) were then applied as above. After further washing, slices were mounted with VectaShield between coverslips as above. Catenary culture explants (see below) were fixed and labelled similarly with QCPN (quail marker, 1/50, mouse IgG, DSHB) and anti-SoxE antibody, with goat anti-mouse IgG: Alexa 488 and anti-rabbit IgG: Alexa 594 (Molecular Probes/InVitrogen). For antibody binding controls, pre-immune rabbit IgG (Jackson Immunoresearch) and mouse IgG CSAT, which does not bind to fixed antigen, were used.

### Neural Crest and Aneural Gut Catenary Cultures

Three types of quail NCC donors were used in tissue and cell culture assays.

1. Isolated neural tubes as NCC donors were obtained from vagal and trunk axial levels at two stages relative to NCC emigration. These were termed *E1.5 vagal* (i.e. vagal neural tube from somite levels 2–6 from 6–10 somite embryos) and *E2 somitic trunk* (i.e. 5 somite lengths of neural tube from the most caudal somitic region of 17–22 somite embryos). These two axial levels represent NCC at the time of EMT and onset of migration. *E2 pre-somitic trunk (*i.e. 5 somite lengths of neural tube from the most caudal pre-somitic region of 17–22 somite embryos) and *E1.5 pre-somitic trunk* (i.e. 5 somite lengths of neural tube at estimated somite level 10–15) were also used, representing earlier premigratory stages.

Tungsten needles were used to excise the neural tube plus paraxial tissues. These tissue segments were placed in 2 mg/ml dispase II (Roche, USA) for 20 min at 37°C, then each neural tube was isolated from all surrounding tissues [55]. Neural tubes were washed three times in Ham’s F12 with 10% FCS briefly to remove the enzyme.

1. *E1.5 vagal paraxial* donors (i.e. intact neural tube and all surrounding paraxial tissues: somites, ectoderm, endoderm) were dissected from somite 2–6 level of 6–10 somite age embryos without dispase.
2. *E4.5 midgut* rostral to the umbilicus and containing endogenous ENCCs were dissected from embryos using tungsten needles without dispase.

Individual NCC donors were placed on Millipore filter paper (HA type, black) support, and put in 3 cm plastic dishes, then cultured in tissue culture media (TCM: 3% fetal calf serum, 1% glutamine, 1% pen/strep in F12) for 0 hr or 2 days, before being placed in contact with the rostral end of a piece of *aneural* chicken gut comprising distal (post-umbilical) midgut, ceca and hindgut of E4.5 (HH25) chicken. These were established as catenary cultures, with the central chicken gut segment unsupported to preserve its tubular structure [56]. Culture times ranged from 2 to 6.5 days.

For different conditions (detailed in results) soluble reagents were added to the NCC donor media either before placement with the gut or during culture, at the following concentrations: 10 µM all-trans-RA (Sigma, USA), 10 µM Am80 synthetic retinoid (Wako Chemicals USA, Inc). RA was also tested at 1 µM (N = 4), 5 µM (N = 3), 20 µM (N = 2) and 26 µM (N = 2), with similar results to 10 µM. The RAR inhibitor CD 2665 (Tocris Bioscience, USA) was used at 800 nM, 1 µM and 2 µM.CD 2665 was also tested at 20 (N = 3), 200 nM (N = 3), and 300 nM (N = 3) and with no visible effect (results not shown).

E1.5 vagal neural tubes were isolated as described above and cultured on black Millipore filter paper in either 10 µM RA or TCM for 2 days, then in RA-free TCM for 2.5 days. Live cells were then revealed by 15–30 min exposure to calcein AM (1/2000, Molecular Probes/InVitrogen, OR) before fixation in 4% PFA.

### Scoring Criteria of NCC Migration in Aneural Gut

For evaluation the aneural gut was divided into five segments proximo-distally. The approximate length measurements for these segments were: post-umbilical midgut 1 = 100 um, post-umbilical midgut 2 = 100 um, ceca = 300 um, hindgut 1 = 200 um, hindgut 2 = 200 um (total length about 0.9 mm). Four criteria were scored to assess colonisation by ENCC. 1. Each segment of recipient gut was given a subjective score 0–4 based on the density of NC cell colonisation; 0 = no invasion; 1 = few sparse cells; 2 = moderate number of cells sparsely distributed; 3 = many cells (typically in chains); 4 = large number of cells (in dense chains and aggregates) (examples are shown below). Representative images were used as a reference to ensure consistency in these scores. These results were averaged for each segment and are presented in Figures below as in [57]. 2. To estimate ENCC numbers, QCPN+ cells were either counted or, for larger numbers (>over 50), a patch of 50 cells was counted and its area measured, then multiplied by the total area of colonised region to give an approximate cell number. 3. The length of intestine colonised was categorised by the location of the most distal NCC in the chick aneural gut segment**.** 4. Each segment was assessed as to whether the ENCC were in grouped (ie. as chains or aggregates), or as apparently single cells.

### Neural Tube Cultures and Proliferation and Apoptosis Assays

Dissected E1.5 quail vagal neural tubes (somite levels 2–6) were cultured on fibronectin-coated (20 µg/ml, 2 hr; Sigma, Australia) 3 cm plastic dishes non-TC dishes (Sarstedt, Australia) in media (500 µl) for 1 hr to promote initial cell-substrate attachment, then were cultured in 1 ml TCM [55]. Soluble reagents were added as detailed in results. Neural tube culture time ranged from 1–7 days. For proliferation/death assays, neural tubes were cultured for 22 hr, then BrdU (1 µl/mL, Abcam) was added to the media for 1 hr prior to 4% PFA fixation. The cultures were then antibody labelled. Primary antibodies targeting BrdU (mouse IgG, 1/100, Amersham) were used. To assess apoptosis, antibody to activated Caspase 3 (rabbit IgG, 1/100, R&D) was used. Secondary antibodies were used as above. To calculate average cell count percentages BrdU+ or Caspase+ cell numbers were divided by DAPI+ cell numbers and multiplied by 100. Each explant was sampled at 2–3 regions (20x field) of DAPI+ neural crest outgrowth (approximately 100–500 cells per field). Two-tailed t-tests for groups of unequal variance were performed to determine statistical significance of differences between groups.

### FACS Sorting for Ret of Neural Tube Cultures Exposed to RA

For vagal level NCCs, E1.5 quail neural tubes were dissected as above. For trunk level NCCs, E2 quail (approximately 20 ss) neural tubes were dissected at somite levels 14–19. Neural tubes were placed on fibronectin-coated dishes and grown in TCM supplemented with 10 µM RA. After 3 days, cultures were washed briefly in F12, then resuspended in 400 µl of 0.5% (w/v) Dispase II (Roche, Germany) and 0.05% (w/v) Collagenase (Worthington, USA) at 37°C and titrated regularly. After 35 min, 1 mM EDTA was added and cells incubated for a further 10 minutes. Single cells were then washed and fixed in 1% PFA for 10 min. Permeabilisation was achieved by treatment with 0.1% Triton X-100 and 1% horse serum. Cells were stained in combination with mouse HNK1 (1/20,MCRI, Australia) and rabbit c-Ret (1/100, IBL, Japan [58]) antibodies in PBS with 1% horse serum and 0.1% Triton X-100. Cells were washed and then stained with goat anti-mouse: Alexa 647 conjugated antibody and goat anti-rabbit: Alexa 488 conjugated antibody (both Invitrogen, USA). Cells were detected for florescence using a MoFlo cell sorter (MoFlo, USA) and analysed with Summit software (Dakocytomation, USA).

### Microscopy

Specimens were imaged with an Olympus IX70 microscope (Olympus Optical Co., Tokyo, Japan) with selective AMCA, FITC and Texas Red filters. Images were recorded using Image-Pro Plus 4.5 (MediaCybernetics, Silver Spring, MD, USA). Image-Pro–Analyser 6.1 (MediaCybernetics) was used for analysis. Confocal images were acquired with a Leica CLSP confocal microscope.

### Ethics Statement

This study was approved by the Royal Children’s Hospital Animal Ethics Committee, permits A596 and A650. This study using early stage avian embryos is deemed non-reportable.

## Results

### ENCCs form Chains *in vivo* at Initial Entry to the Foregut

**Figure 1:**
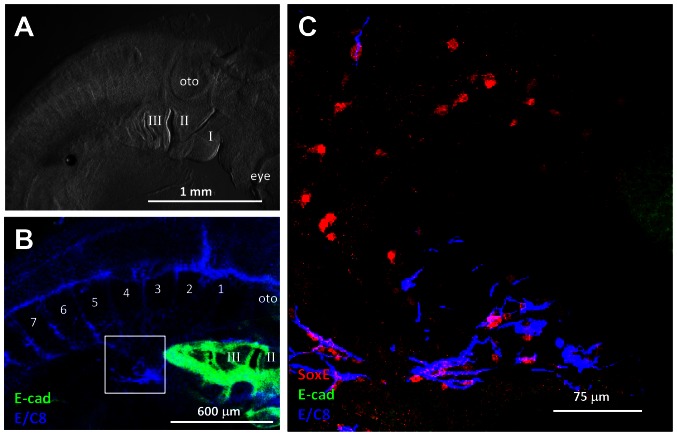
VNCCs form chains on arrival in foregut mesenchyme. **A.** Sagittally sliced E2.5 quail embryo viewed from the medial aspect, with rostral to the right. Oto = otocyst; I = branchial arch I (mandibular division); II = branchial arch II; etc. (Light field microscopy) **B.** Pharyngeal endoderm of same specimen revealed by E-cadherin antibody labelling. The endoderm of the narrowed foregut caudal to the pharynx is not present in this half-specimen. Axons detected by E/C8 antibody converge ventrally from vagal somite levels (somite 1, 2 etc.), lateral and ventral to the foregut, in the boxed area. (Confocal microscopy) **C.** Boxed region in B showing chains of SoxE+ve nuclei of ENCC in the mesenchyme of the foregut. In the central part of this image, ENCC and axons are not associated; axons are more laterally placed and excluded from this confocal optical section. Ventrally (this image) and more laterally, NCC and axons are associated.

Sagittal slices of E2.5 quail embryos (HH16–18; N = 14) stained with antibodies to SoxE to label NCCs and E-Cadherin to label foregut endoderm displayed ENCC in spiralling chains usually 1–2 cells wide within the mesenchyme of the narrow foregut immediately caudal to the wide pharynx from the earliest stages of colonisation (Figure 1). Vagal axon tracts coursing ventrally towards the foregut were readily recognised by E/C8 immunoreactivity especially in the slightly older specimens (Figure 1B). SoxE+ NCC accompanied these but adjacent to the foregut these axons were placed lateral to the ENCC (Figure 1C). Therefore it is likely that ENCC first colonise the foregut mesoderm as cell chains before the arrival of axons.

### ENCCs from E4.5 Midgut Colonise Gut Immediately, Prolifically and in Chains

E4.5 quail midgut tissue containing ENCCs were placed at the rostral end of E4.5 aneural chicken gut for 2 days in catenary organ culture. The QCPN+ (i.e. quail origin) and SoxE+ (i.e. NC cells-derived) ENCCs migrated in high numbers (approximately 1000 cells) and colonised as far as the hindgut, approximately 650–700 µm linearly (N = 15). Assuming a cylindrical shape (d = 120 µm) and a single ENS layer, this corresponds to an ENS cell density of about 40–50 cells/0.01 mm^2^; similar to QE6 midgut *in vivo* (71±4.5 cells/0.01 mm^2^; Zhang personal observation). In these and other catenary cultures, the range of scores for ENCC numbers between specimens within each experimental condition was highly consistent proximally (in midgut 1 and 2 segments), with scores becoming more variable distally (in cecal, hindgut 1 and hindgut 2 segments). This distal variability between specimens involved the attainment (or not) of a segment by the wavefront ENCC, and the density of ENCC in the attained distal segment. Colonisation of the extreme distal hindgut was always incomplete but this is delayed even *in vivo* [4], [59].

**Figure 2:**
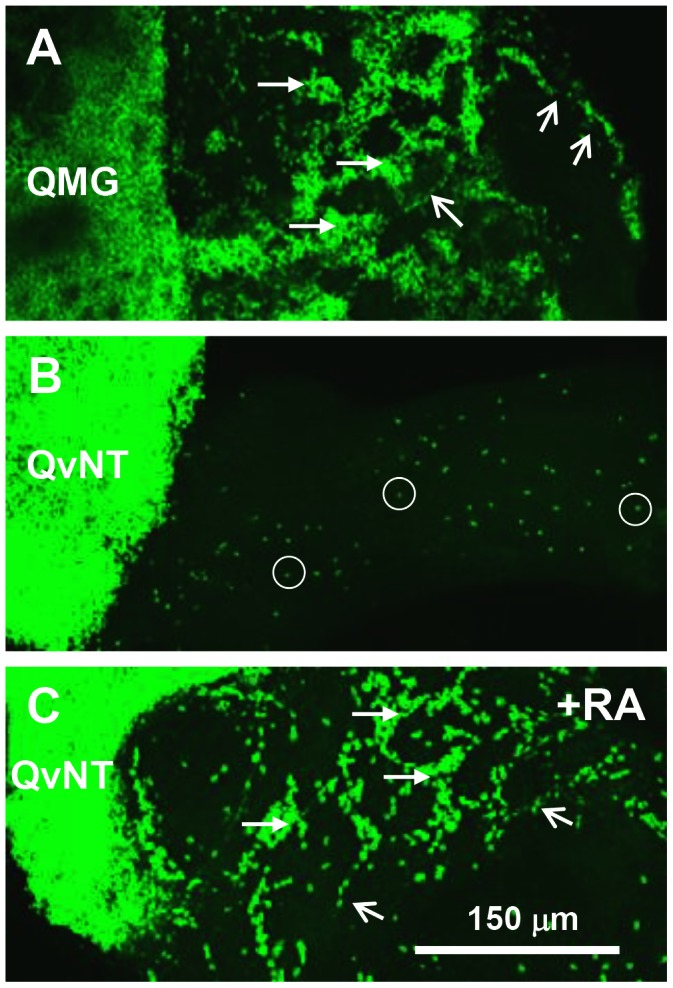
Catenary co-cultures reveal different colonising ability of NCCs. **A.** Quail E4.5 midgut (QMG) as ENCC donor co-cultured with E4 aneural chicken gut for 2 days. Quail cells are labelled in green with QCPN antibody. NCCs colonise the aneural gut in high numbers and in chains (open arrows) and aggregates (filled arrows). **B.** Quail E1.5 vagal neural tube (QvNT) VNCC donor after 2 days culture, then 3 days co-culture with a segment of aneural chicken gut. Quail cells are labelled in green. NCCs (examples circled) colonise the tissue poorly as sparsely distributed cells**. C**. Quail E1.5 vagal neural tube (QvNT) NCC donor after 2 days culture in 10 µM retinoic acid, then 3 days co-culture with aneural chicken gut. Quail cells are labelled in green. NCCs enter the tissue in high numbers and are distributed as chains and aggregates.

**Figure 3:**
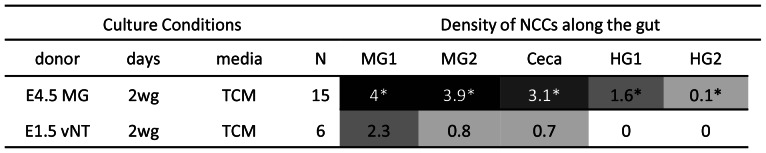
E1.5 VNCC donors are poor at colonising aneural gut in catenary cultures, relative to E4.5 ENCC donors. Colonisation performance (cell number and chain formation) of QCPN+ cells was quantified on a five point scale (0 = nil to 4 = dense chains and aggregates, in 5 gut regions (MG1, MG2, Ceca, HG1, HG2). Averages are shown. The following shading system was applied: 0.0, white; 0.1–1.4, light gray; 1.5–2.4, medium gray; 2.5–3.4, dark gray; 3.5–4.0, black. * denotes cells in chains or aggregates. E = quail embryonic day, vNT = vagal neural tube, MG = midgut, wg = with gut.

The ENCCs arranged themselves in chains up to 4 cells in width, which extended even to the wavefront cells. This mode of migratory cell morphogenesis is a well described property of early ENS development [6], [7], [60]. These ENCCs show colonisation speed, distance, formation and numbers essentially identical to *in vivo* gut colonisation [11]. We use this qualitative and quantitative outcome to represent optimal gut colonisation ability (Figures 2A, 3).

### VNCCs Neither Colonise Gut Immediately in Large Numbers, nor Form Chains

Vagal level (s2–6) E1.5 (7–11 ss) quail neural tubes containing pre-migratory VNCCs were cultured with chick aneural gut for 2 days (N = 6); these resulted in poor colonisation by the criteria we used. QCPN+/SoxE+ VNCCs migrated into the aneural tissue in low numbers (approximately 50–150 cells), with the most distal cell only penetrating a short distance (approximately 150 µm), that is, rarely proceeding past the distal midgut 2 (Figure 3). The NCCs were sparsely but relatively evenly arranged, and they did not form chains. However, when cultured in fibronectin-coated plastic dishes, E1.5 vagal neural tubes produce large numbers of migratory NCCs, with 1000–2000 migrating cells by 1 day *in vitro* (N>50). However under these *in vitro* conditions the VNCCs never formed chains (see also [18], [61], [62]). We conclude that the VNCC, while migratory *per se*, initially lack some ability that enables them to efficiently colonise gut mesenchyme as chains of cells.

### VNCCs Improve Colonisation with Increased Time of Contact with Gut

**Figure 4:**
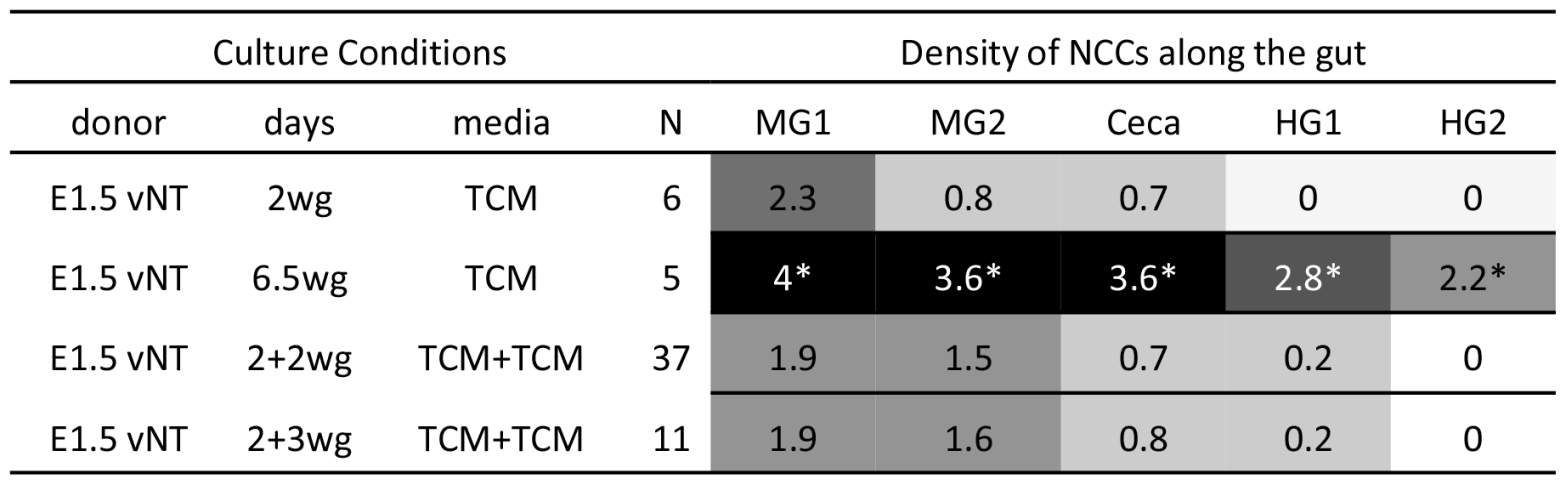
Longer time of VNCC in contact with gut in culture, but not longer time in culture alone, improves colonisation of aneural gut. Abbreviations as in Figure 3.

One difference between the two NCC donor systems is NCC age (E1.5 vs E4.5) at commencement of the 2 day aneural gut colonisation assay. Therefore we increased the culture time of neural tube-derived VNCCs in the presence of aneural gut tissue. Vagal level quail neural tube cultured with aneural gut for 6.5 days produced increased colonising cell numbers and often chain-like cell distribution (N = 5) (Figure 4). This improvement was discernible by 4 days culture (N = 15, not shown). However, this result was variable in form as increased time with gut tissue did not always produce chains, as invariably occurred with E4.5 midgut donor cultures. Rather, the ENS cells sometimes formed patches or aggregates. This resembled later stage ENCC development, several days after initial colonisation of the gut, as ganglia form [8], [63]. We conclude that the VNCC gain some quality which that enables them to efficiently colonise gut mesenchyme as chains or groups of cells, and this acquisition requires either time from onset of migration (that is, from their epithelial-mesenchymal transition or EMT), or time in contact with gut tissue.

An example of a dense colonisation with a score of 4 is shown in Figure 2A.

### Ability to Colonise Gut is not Conferred on VNCCs by Time After EMT

We tested the notion of a solely time-dependant mechanism producing this colonisation improvement. In normal development, the VNCCs would have 1–2 days from EMT at the dorsal neural tube before reaching and entering foregut. We showed above that *in vivo* these cells form chains in the foregut at this stage. Vagal level quail neural tubes were cultured alone for 2 days, then the aneural gut was added for 2 days (termed 2+2 cultures, N = 37). Cells migrated into the aneural tissue in low numbers (approximately 50–200 cells), and were distributed sparsely and did not form chains. Typically the most distal cells was found short distance, about 250–500 µm) into the cecal region, but occasionally cells were scattered into the hindgut. When cultured for 2 days alone, but the time with aneural gut was increased to 3 days (2+3; N = 11), NCC distribution was similar (i.e. sparse arrangement, no chains, and approximately 80–250 cells, (Figure 2B, Figure 4). Figure 2B shows moderate NCC colonisation density (score of 2).We conclude that the VNCC acquire some capacity from gut tissues after prolonged contact that enables them to efficiently colonise gut mesenchyme as chains of cells, and this capacity does not develop spontaneously with time after EMT.

### Ability to Colonise Gut is Conferred on VNCCs by Surrounding Paraxial Tissues

**Figure 5:**
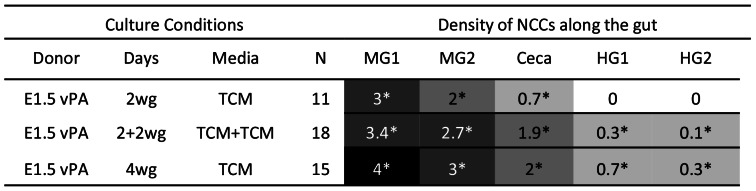
Vagal paraxial tissues improve colonisation of aneural gut by E1.5 VNCCs. Abbreviations as in Figure 3, plus vPA = E1.5 vagal neural and paraxial tissues.

Vagal paraxial donors (i.e. vagal level neural tube/crest with surrounding tissues), were cultured either immediately with the aneural gut for 2 days (N = 11) or cultured 2 days alone and then 2 days with aneural gut (2+2; N = 18). In both cases NCCs were able to enter the tissue in high numbers (approximately 500–800 cells), and form chains generally 2–4 cells in width. The distance of penetration of the most distal NCCs was the only clear difference in the two situations: NCCs in 2+2 day cultures reached the hindgut, while in 2 day cultures they reached the ceca). Vagal paraxial donors cultured immediately with the aneural gut for 4 days (N = 15) showed chained colonisation reaching the distal hindgut, comparable to 2 day E4.5 midgut donor cultures, (Figure 5). We conclude that in less than 2 days the VNCC gain some quality from paraxial tissues that enables them to efficiently colonise gut mesenchyme as chains of cells.

### CRABP-1 is Expressed *in vivo* by VNCCs Near to the Foregut

**Figure 6:**
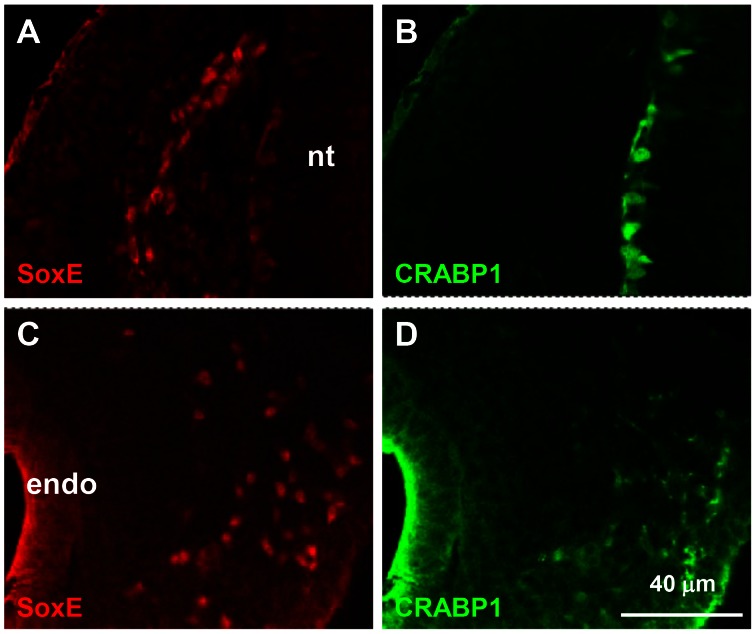
NCC gain CRABP-1 immunoreactivity near and in the foregut. **A, B**. Quail E2.75/HH17 foregut level transverse slice. Dorsally placed SoxE+ vNCC (A) show little CRABP-1 immunoreactivity (B), in contrast to adjacent neural tube tube (nt) cells. **C,D**. At E3/HH20, more ventrally placed SoxE+ ENCC (C) lateral to the foregut endoderm (endo) are also CRABP-1+ve (D). Dorsal somitic mesenchyme (B) shows less reactivity with CRABP-1 antibody than does foregut mesenchyme (D).

Confocal analysis of two somite wide transverse slices through the vagal level at E2.75–3 (N = 13 embryos) showed little or no CRABP-1 immunoreactivity in SoxE+ cells near the neural tube (Figure 6A,B). In contrast CRABP-1 immunoreactivity occured in SoxE+ NCC in and near the foregut mesenchyme in the same preparations (Figure 6C,D). There was also low level staining of gut mesenchyme by the CRABP-1 antibody that was absent in dorsal somitic mesenchyme. This, and published data referred to in the Introduction, suggests that RA signalling appears in VNCC after a period of migration towards and in the gut. RA signalling is therefore a candidate mediator of the effect of paraxial tissue on VNCC to enable efficient enteric colonisation.

### RA Improves Ability of VNCC to Colonise Gut in Chains

**Figure 7:**
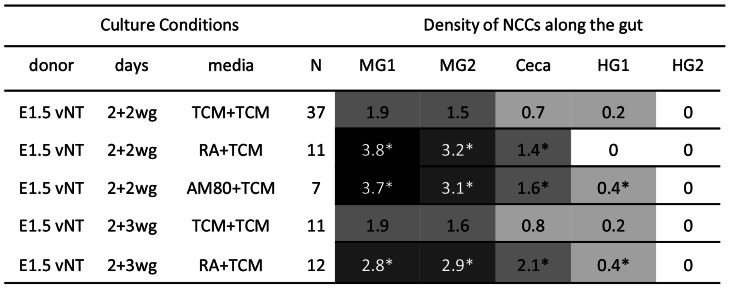
RA improves gut colonisation of aneural gut by E1.5 VNCCs. Abbreviations as in Figure 3, plus RA = retinoic acid; AM80 = synthetic retinoid which is not able to be processed by CYP26A1.

Vagal level quail neural tube cultured in 10 µM RA for 2 days, then cultured with the gut for 2 days (2+2 culture) produced near optimal colonisation qualitatively. The NCCs were consistently able to enter the tissue in high numbers (approximately 600 cells per specimen) and formed chains (N = 11) (Figure 7). These chains were approximately 1–2 cells in width and appeared comparable to E3 foregut colonisation *in vivo* (Figure 1), and to E1.5 vagal paraxial donors cultured with aneural gut (Figure 5). The most distal NCCs migrated similar distances in the presence or absence of RA in 2+2 day cultures (up to 500 µm, reaching the ceca/hindgut level). The major difference was the RA treated donors produced much more abundant and densely distributed ENCCs in chains. A further 1 day organ culture with the gut (2+3 days, N = 12) resulted in these chains becoming even more pronounced (3–4 cells wide) and extending further into the hindgut, identical to the E4.5 midgut NCC donor cultures described above (Figure 2C, Figure 7).

### CYP26A1 Processing of RA and RARβ are not Required for ENCC Chain Formation

Am80 is a synthetic retinoid that cannot be processed by the RA-catabolising-enzyme CYP26A1. CYP26A1 expression parallels the migrating NCCs during development [30]. E1.5 vagal neural tube exposed to 10 µM of Am80 for 2 days, then placed with aneural gut for 2 days (N = 7)(Figure 7), resulted in ENCCs colonising the aneural gut in chain formations, identical to RA-exposed equivalents. These results suggest that CYP26A1 metabolism and products (including the active compound 4-oxo-RA) are not required for the induction of chain migration and colonisation observed in RA cultures (Figure 7). Additionally, Am80 does not signal through through RARβ [64], suggesting that signalling via this receptor is not necessary for ENCC chain migration.

### RA Receptor Inhibitor Disrupts Chain Formation and Gut Colonisation

**Figure 8:**
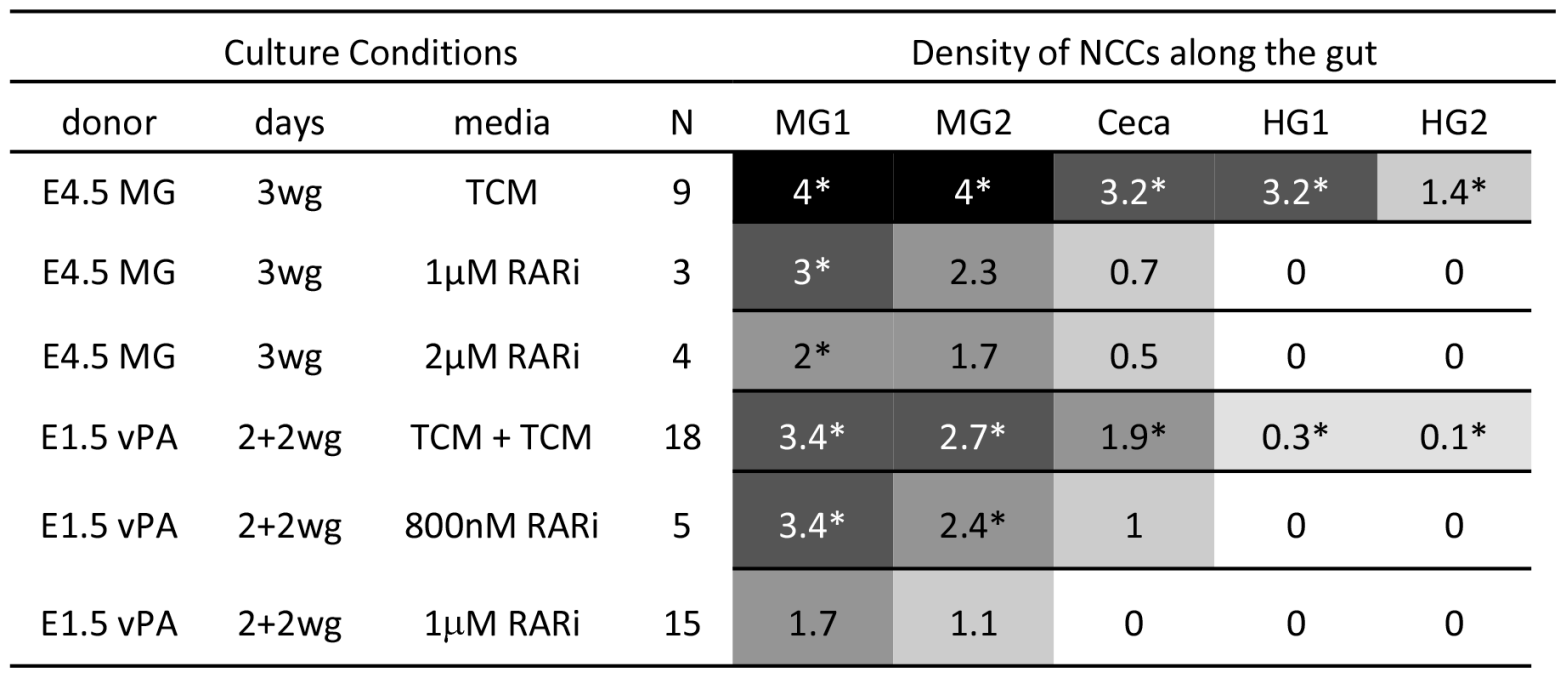
RAR inhibitor disrupts colonisation of aneural gut. Abbreviations as in Figure 3, plus RARi = RAR inhibitor CD 2665. In the 2+2 series, the RARi was present only in the first 2 day period.

E4.5 midgut ENCC donor cultured in 1–2 µM RAR inhibitor CD 2665 with aneural gut for 3 days produced disrupted chain formation (N = 7)(Figure 8). ENCC were fewer than in TCM controls (approximately 500–600 cells reaching 300–400 µm to the ceca; compared with more than 1000 cells reaching 650–700 µm to the hindgut). Although some ENCCs in CD 2665-treated cultures were located close together, suggesting cell-cell contacts, chains were not extensive compared to TCM controls, and wavefront NCCs in particular were in single-cell arrangement.

Vagal level quail neural tube with paraxial tissues still attached cultured in 1 µM RAR inhibitor CD 2665 for 2 days, then cultured with aneural gut in TCM for 2 days produced poor colonisation, with fewer cells migrating a shorter distance into aneural gut tissue than TCM controls (approximately 300–400 cells reaching midgut 200 µm) (N = 15) (Figure 8). Some chains were present (generally 1–2 cells wide) but many single, unevenly and distantly spaced NCCs were present, particularly at the wavefront. These results taken together indicate both an initial and an ongoing role for RA in ENS colonisation. This inhibitory effect of CD 2665 on enteric colonisation was apparent at high concentrations of the inhibitor, (above 800 nM), but little to no affect was seen at 800 nM (N = 5) or less (N = 9, not shown).

The RAR inhibitor CD 2665 is more effective against RARγ and RARβ than RARα signal transduction [65]. Since little or no effect was seen in the present assays at 800 or less, concentrations equal and greater than those previously found effective against RARγ and RARβ, this suggests that RARγ and RARβ are not required for normal ENCC migration, and therefore that RARα is necessary for the response to RA by VNCCs.

### RA and RAR Inhibitor affect Proliferation and Apoptosis in VNCC *in vitro*

**Figure 9:**
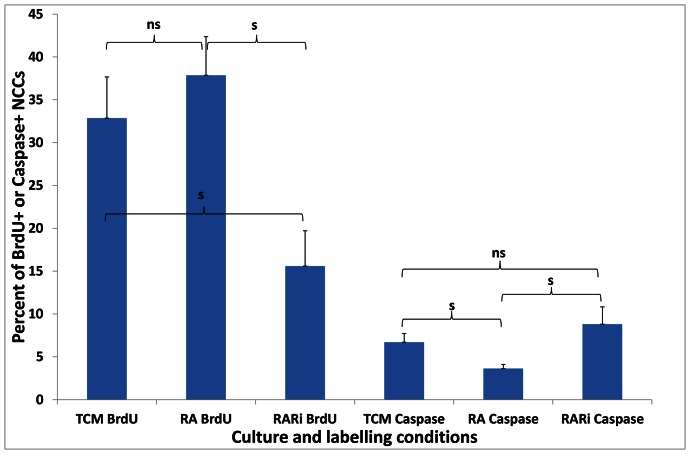
RA signalling agonist and antagonist alter the percentages of BrdU positive and caspase-3 positive VNCCs in neural tube/NC cultures. Percent (±s.e.m.) of positive labelling is listed on the Y-axis, and culture and labeling conditions are listed on the X-axis. Number of explants in: TCM N = 7, 10 µM retinoic acid (RA) N = 21, 1 µM RAR inhibitor CD 2665 (RARi) N = 9. s = significant differences (p≤0.05); ns = not significant difference.

BrdU+staining was used as a measure of proliferation, and Caspase-3 staining was used as a measure of apoptotic cell death in conventional NCC cultures. RA administration to vagal neural tube explants cultured for 2 days on fibronectin coated dishes did not result in a significant difference in proliferation in VNCC outgrowths as detected by BrdU antibody staining following 1hr BrdU exposure, and slightly but significantly reduced the apoptosis rate as detected by caspase-3 labelling (Figure 9). Addition of 1 µM RAR inhibitor CD 2665 to these cultures resulted in a highly significant decrease in BrdU staining. An increase in apoptosis in CD 2665 treated cultures, compared to TCM, did not attain significance (Figure 9). The opposing effects of RA and RAR inhibitor CD 2665 in the catenary cultures may therefore reflect these opposite effects on NCC proliferation and survival. Cell death levels were generally low, suggesting that altered proliferation rate (which was significantly lower with RAR inhibitor) is the more likely contributor for the differences in colonisation in catenary cultures.

### RA induces VNCC Aggregation *in vitro*

RA application was able to induce VNCC aggregation *in vitro* on low adhesion, non-smooth substrate (Millipore filter paper). E1.5 vagal neural tubes were dissected and cultured in 10 µM RA on Millipore filter paper for 2 days, then in RA-free TCM for 2.5 days. Cells were then detected before fixation with calcein AM. VNCCs migrated out from the neural tube 400–500 µm radially from the explant and formed aggregates of approx 3–6 cells width on the substrate in RA treated explants (N = 7, compared to control explants, N = 6). In contrast, without RA the VNCC outgrowth was a uniform cell layer. On higher adhesion substrate (fibronectin-coated plastic dish) where NCC adopt a highly flattened shape, 10 µM RA exposed (N = 10) and TCM control VNCC migrated out onto the substrate in a typical monolayer outgrowth with no sign of aggregation in either condition.

### RA Increases Ret Expression *in vitro*

**Figure 10:**
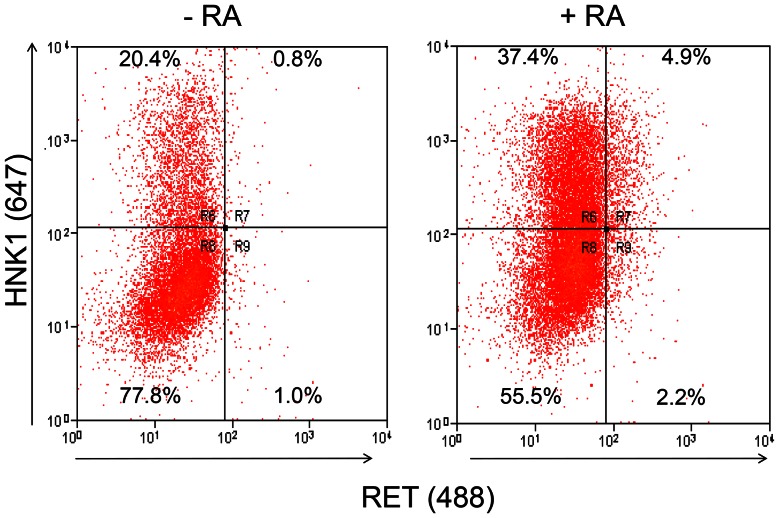
Induction of Ret following RA treatment of E1.5 neural tube cultures. E1.5 neural tube/NC explants were cultured for 3 days with/without 10 µM RA (pooled from 45 and 42 explants respectively) and analysed by FACS for HNK1 expression (fluorescence emission at 647 nm) and RET expression (fluorescence emission at 488 nm). The application of RA almost doubled the proportion of HNK1+ cells and increased the proportion of HNK1+/Ret+ cells by approximately 6-fold.

FACS based on labelling cells for HNK1 and c-Ret antibodies indicated that E1.5 vagal neural tube cells and NCCs (pooled from N = 42 explants) cultured in TCM for 3 days have few Ret immunoreactive cells, practically all of which were HNK1 labelled. When cultured with 10 µM RA for 3 days (pooled from N = 45 explants), similar cultures showed a higher proportion of cells were HNK1 immunoreactive, and of these, an even higher proportion (6-fold increase) also labelled for Ret (Figure 10).

### RA does not Improve Ability of Trunk NCC to Colonise Gut

**Figure 11:**
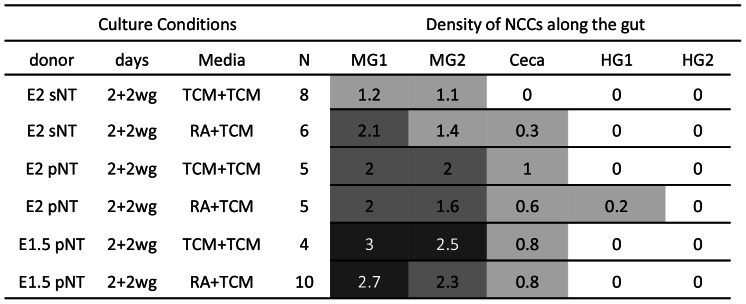
Trunk NCC donors are poor at colonising aneural gut and RA does not improve this. Abbreviations as in Figure 4, plus sNT = somitic level trunk neural tube, pNT = pre-somitic level trunk neural tube.

Trunk-level NC is quantitatively poor at colonising aneural gut [61]. Trunk neural tube with NCCs, including E2 somitic level (N = 8), a developmental age equivalent to the E1.5 VNCC donors, and developmentally younger pre-somitic level trunk neural tube at E2 (N = 5) and E1.5 (N = 4) were cultured as controls for 2 days then with aneural gut for 2 days. Similar trunk level NCC donors (N = 6, 5 and 10 respectively) were exposed to 10 µM RA for 2 days before combination with aneural gut for 2 days. Trunk NCC colonisation was similarly poor in the RA and control cultures. Cells entered aneural gut in low to moderate numbers, and in nearly all cases migration reached no further than the ceca, and cells were always sparse and did not form chains (Figure 11). Thus RA had little effect on trunk NCC in regard to enteric colonisation ability.

## Discussion

### Important Events for ENS Formation Prior to VNCC Arrival in the Foregut Involve RA Signalling

Combination chorio-allantoic membrane grafts of avian aneural gut with vagal neural tube bearing premigratory NCC produced full ENS colonisation of the former. Paraxial tissues between the dorsal neural tube and the foregut, through which VNCC migrate initially, were not included in these grafts. It was concluded at the time that these paraxial tissues had no role in ENS formation [66], [67].

Recently, Zebrafish studies have demonstrated the importance for ENS development of morphogen signalling (in this case Hedgehog) during this early migration phase [68]. In work presented here we show that paraxial tissue encountered by avian VNCC is of importance in allowing efficient colonisation of the gut, and suggest this is mediated via RA signalling.

### Differences between VNCC and Trunk NCC in ENS Potency may Involve Differential Responses to RA

In the chorio-allantoic membrane grafts mentioned above, a full ENS formed only when the neural donor was of vagal or cranial origin [61], [67]. This shows that, even prior to migration, trunk NCCs have diminished competence to form a quantitatively complete ENS, in contrast to vagal NCC and indeed even to the cranial NCC which are not fated to form ENS. This suggests that trunk NCCs before EMT and migration are unable or less able to respond adequately to environmental cues such as RA which otherwise favour ENS formation. We have shown that the VNCC RA response includes upregulation of Ret, and previous studies suggest trunk NCC show lower levels of endogenous Ret expression, and artificial upregulation of Ret via electroporation confers improved enteric colonisation ability [69].

### The Colonisation-promoting Effect of VNCC/ENCC Proliferation Induced by RA Requires Tissue Factors

Using neural tube as VNCC donors, there were clearly many more ENCC in catenary cultures with addition of RA. We suggest that *in vivo* and in tissue microenvironment in organ culture, RA increases VNCC/ENCC proliferation. Increasing ENCC numbers is known to be necessary for gut colonisation [11], [13], [61], [70]–[72]. However, we did not detect a significant increase in proliferation with exogenous RA in our neural tube/NCC *in vitro* assay, though the decline in proliferation with RA receptor blockade suggested that this pathway was active: it may be that the neural tube in these cultures provided endogenous RA. A decrease in VNCC cell death was also seen in these assays *in vitro*, but we suggest this is unimportant *in vivo*, where cell death is very low normally. It may be that VNCCs require RA and other factors from tissues (eg. GDNF) not available in the conventional *in vitro* assays to increase proliferation, and in our catenary cultures, GDNF may be supplied by the E4.5 recipient gut, or the variety of tissues (somite, ectoderm, endoderm, notochord) included in E1.5 vagal level paraxial tissue donors.

### The Chain Migration/Aggregation Effect of ENCC Induced by RA does not Require Tissue Factors

Early migrating NCCs form chains in the cranial and somitic paraxial mesenchyme [73], [74], but evidently this capacity is not automatically extended to the gut mesenchyme. Therefore we additionally propose that RA also promotes cell adhesion changes to allow stereotypical chain migration and, later, aggregation, specific for the gut mesenchymal microenvironment. One candidate effector molecule is the adhesion molecule L1CAM, since this is expressed on early chain-migrating avian ENCC [8], [63], and genetic and antibody perturbation experiments indicate that it is important in ENCC chain maintenance in the hindgut of the mouse [58].

There are a number of other molecules of interest in the migratory and ganglion morphogenesis of ENCC. Avian NCC adhesion, migration, and survival *in vitro* is facilitated by α4-β1 integrin [75]. NCCs express β1-integrins and in mouse, NCC-specific β1-integrin-null embryos, ENCCs show retarded migration at the ceca, then colonise the cecum and proximal hindgut abnormally [76], that is, chain formation is disrupted. N-cadherin, a cell-cell adhesion molecule on ENCC [8], [63] is also a molecule of interest, with double knockouts of N-cadherin and β1-integrin in NCC causing severe ENS malformation with altered speed of locomotion and directionality of ENCC in the gut wall [77].

We could not reproduce chain migration by VNCC in *in vitro* assays, but RA did induce cell aggregation *in vitro* on Millipore filter paper substrates. This suggests that increased cell-cell adhesion *in vitro* may require RA and not tissue factors like GDNF (unlike the proliferation response discussed above), albeit in the context of unusual substrate biophysical properties.

### Conclusion

We propose that VNCCs normally experience RA secreted from the somites [27], [28] which they migrate through (somite levels 3–7) or near (somite-levels 1 and 2) *en route* to the foregut, and/or later from the foregut itself [26], [27], [30], [36]–[40], [78], [79]. We further propose that in response the VNCCs increase Ret expression, allowing them to respond to GDNF being secreted by the gut mesenchyme [14]. This produces the known effects of GDNF on ENS cells: increased ENCC survival, migration, proliferation and differentiation [17], [18], [22], [80]. We also suggest that RA promotes cell interactions enabling chain formation. We conclude that RA signalling is required for efficient initial invasion of the foregut as well as for continued ENS colonisation, morphogenesis and differentiation [16], [81]–[84].

Considerable interest is being displayed in the possibility of creating NC stem cells for treatment of enteric neuropathies [85]. Given the similarities of stem cell biology to normal embryonic developmental processes, and given the conservative nature across different species of ENS development, our previous developmental studies led us to propose that to enable NC stem cells to form an ENS efficiently requires specific vagal or cranial positional information which is normally acquired before EMT [61]. The present study proposes that NC stem cells for this purpose would also require the equivalent of post-EMT changes in the form of RA exposure, and the Zebrafish studies suggest that post-EMT Hedgehog signalling would also be important at this stage [68].

### Ethics Statement

This study was approved by the Royal Children’s Hospital Animal Ethics Committee, permits A596 and A650. This study using early stage avian embryos is deemed non-reportable.
